# Shared Brain Lateralization Patterns in Language and Acheulean Stone Tool Production: A Functional Transcranial Doppler Ultrasound Study

**DOI:** 10.1371/journal.pone.0072693

**Published:** 2013-08-30

**Authors:** Natalie Thaïs Uomini, Georg Friedrich Meyer

**Affiliations:** 1 Department of Archaeology, Classics and Egyptology, University of Liverpool, Liverpool, United Kingdom; 2 Department of Experimental Psychology, University of Liverpool, Liverpool, United Kingdom; University of Oxford, United Kingdom

## Abstract

**Background:**

The popular theory that complex tool-making and language co-evolved in the human lineage rests on the hypothesis that both skills share underlying brain processes and systems. However, language and stone tool-making have so far only been studied separately using a range of neuroimaging techniques and diverse paradigms.

**Methodology/Principal Findings:**

We present the first-ever study of brain activation that directly compares active Acheulean tool-making and language. Using functional transcranial Doppler ultrasonography (fTCD), we measured brain blood flow lateralization patterns (hemodynamics) in subjects who performed two tasks designed to isolate the planning component of Acheulean stone tool-making and cued word generation as a language task. We show highly correlated hemodynamics in the initial 10 seconds of task execution.

**Conclusions/Significance:**

Stone tool-making and cued word generation cause common cerebral blood flow lateralization signatures in our participants. This is consistent with a shared neural substrate for prehistoric stone tool-making and language, and is compatible with language evolution theories that posit a co-evolution of language and manual praxis. In turn, our results support the hypothesis that aspects of language might have emerged as early as 1.75 million years ago, with the start of Acheulean technology.

## Introduction

Complex tool-making and language are two areas that set humans apart from other animals [1]. The emergence of unique toolkits based on the physical effects of striking two stones together occurred by c. 2.5 Mya (million years) ago [2], [3], or possibly even earlier [4]. In contrast, estimates for the emergence of language range from *Homo erectus/ergaster* [5] at 1.89 Mya to Upper Palaeolithic modern humans [6] at 50,000 years ago. The crucial question under debate is to what extent the evolution of stone tool-making capacities and linguistic capacities were aligned [7], [8]. The popular theory that both skills co-evolved in the human lineage [9]–[11] rests on the hypothesis that both skills share underlying processes and neural systems, but there is little empirical evidence for this [12]. Direct evidence that both skills draw on common brain areas or result in common brain activation patterns would provide compelling support for this argument.

Language and stone tool-making have so far only been studied separately using a range of neuroimaging techniques and diverse paradigms [13]. Both share conceptual similarities, such as the need for structured and hierarchical action plans [9], [14], [15] to be successfully executed. There is considerable co-development of tool-use and language in human children [1], [10]. Language dominance predicts the laterality of temporal and spatial movement representations in ideomotor praxis: the ability to imagine or act out motor actions that rely on semantic memory [16]. Most importantly, there is a potential for substantial overlap in the neural circuits activated during tool-use [17], [18] and language [19], including action planning and action observation [20].

**Figure 1:**
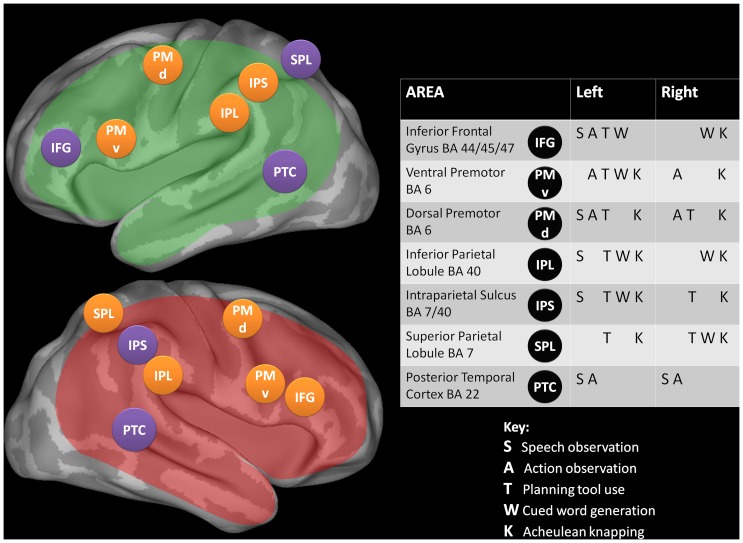
Brain regions activated during speech and action observation, tool-use, word generation, and Acheulean knapping. S = speech observation, A = action observation, T = planning tool use, W = word generation, K = Acheulean knapping. The table shows potential overlap in the neural networks used in all five tasks. The posterior temporal cortex is used exclusively for observation. Fronto-parietal brain areas activated by both cued word generation *and* knapping are highlighted in orange. Cortical areas supplied by the MCA (middle cerebral artery) are highlighted in green (left hemisphere) and red (right hemisphere).

Figure 1 shows a schematic diagram of the broad fronto-parietal brain areas that have been implicated in a range of language and action observation, planning and execution tasks [21]–[23]. Previous work has shown that speech (S in Figure 1) and action (A) observation draw on shared networks identified by [20] that are simultaneously activated [24]. Similarly, Acheulean stone tool-making (knapping, K, [23]), and planning tool-use actions (T, [25]) have been reported to activate similar anatomical brain areas. These areas are also used during cued word generation (W, [26]). Some brain areas, notably regions in the posterior temporal cortex (PTC) are selectively involved in observational tasks (S, A), while the fronto-parietal network is active during execution and observation.

Because all of these findings derive from separate studies using disparate methodologies, they identify broad areas that do not necessarily overlap within individual participants. The key question is whether the overlap is functionally relevant. The objective of the present study was to directly compare real-time brain activation patterns for language and stone tool-making (knapping) within one group of participants, using one single neuroimaging technique and experimental paradigm.

We selected Acheulean knapping (K in Figure 1) as the tool-making task and cued word generation (W in Figure 1) as the language task because previous independent studies identified activation for these tasks in overlapping bilateral parietal and frontal cortical sites, marked in orange in Figure 1. The cued word generation task is widely used in clinical language lateralization assessment and provides a well-established set of benchmark data against which to validate our results [27]–[29]. Furthermore, brain activation patterns during stone knapping [23] suggest relatively higher cognitive demands when making Acheulean bifaces compared to Oldowan flakes. Acheulean knapping requires increased visuomotor coordination and hierarchical action organization [30]. The emergence of the Acheulean techno-complex points to a change in the cognitive capabilities for making stone tools before 1 Mya. The extensively worked Acheulean handaxes mark an increased complexity of technological production from Oldowan flaking [12], [31], [32], with longer sequences and sub-sequences of stone tool shaping to achieve a more refined product.

We employ functional transcranial Doppler ultrasonography (fTCD). For a good review of fTCD, see [33]. This portable, non-invasive technique uses Doppler ultrasound to measure blood flow velocity changes in the right and left middle cerebral arteries (MCAs) during language and stone tool production. The MCAs supply the majority of the lateral surface of each cortical hemisphere with the exception of the most superior sections of the frontal and parietal lobes, the occipital cortex, and the inferior part of the temporal lobe [34], as indicated by the areas shaded red (right) and green (left) in Figure 1. The activation of brain areas supplied by the MCA, such as the network shown in Figure 1, causes intra-cranial blood flow velocity (CBFV) changes.

In contrast to most other neuroimaging methods, such as fMRI, which is standard for language tasks, fTCD is not vulnerable to participant motion, so that the vigorous physical action of stone knapping is possible. In contrast to PET (positron emission tomography), which has been used to study stone knapping [35], fTCD offers sufficient temporal resolution to measure rapid changes in cerebral blood flow patterns. The equipment is small and portable. fTCD is thus ideally suited for combining real-time tasks such as language with tasks such as stone knapping which involve subject motion and recording outside the laboratory.

The fTCD technique has been used for language neuroimaging since 1998, providing well- documented and highly replicable baseline results [36], [37], in particular for language lateralization studies. fTCD measures of cerebral blood flow lateralization are highly correlated with alternative measures, for example the relative distribution of fMRI voxel counts in the left and right hemispheres for language tasks [26], [38] and spatial attention tasks [39], [40]. Sabri et al. [41] showed that simultaneously recorded fTCD lateralization data were almost perfectly correlated with PET volume changes and volume-weighted perfusion changes in a (n-back) working memory task. Language lateralization data from fTCD have further been shown to correlate well with unilateral disruption of language functions via either the intracarotid sodium amobarbital procedure (Wada test [42]) or repetitive Transcranial Magnetic Stimulation (rTMS) [43]. The fTCD technique therefore provides reliable data that directly reflect the activation state of brain areas supplied by the artery under investigation.

The cued word generation task was chosen for this study because a wealth of comparison data from fTCD and other imaging methodologies exists for it; the CBFV changes we observe can therefore be directly compared with previous work. Bishop et al. [28] describe the task as the ‘gold standard’ and show highly correlated lateralization indices (LIs) for this task with those obtained for two other language tasks that rely more on syntactic processing: a picture description task and an animation description task, consistent with the view that all three tasks draw on substantially overlapping cortical networks.

Furthermore, a number of studies show that a range of visuo-spatial tasks lead to LIs that are *not* correlated with the standard cued word generation task: Rosch et al. [44] tested visuo-spatial attention, Whitehouse et al. [45] used a visual memory task, and Lust et al. [46] tested participants in a driving simulator. None of these studies found a correlation with cued word generation. Rosch et al. [44] also show that the LI correlation is not affected by task difficulty. These findings are relevant for this study because they mean that neither common attentional processes at the onset of two different tasks, nor obligatory contralateral brain organization for language and visuo-spatial processing, are plausible explanations for correlated brain blood flow changes.

If action planning for tool-making and language draw on shared functional brain structures, we predict that the individual hemodynamic modulation measures for the two tasks should be positively correlated. In other words, individuals who show highly lateralized rapid blood flow changes for language should show a similar response during stone knapping. Specifically, this correlation should occur in the initial planning phase of task execution. The first few seconds of cerebral hemodynamics have been shown to be a good predictor of complex behavior, such as performance in non-routine planning tasks [47].

## Materials and Methods

### Subjects

All participants were experienced stone knappers, recruited at a meeting of the UK Lithic Studies Society and from the Archaeology Department at the University of Liverpool. We report on data from 10 participants (3 female, age range 21–68, mean age 37.7 years). All were healthy and without a history of neurological disorder. Two male participants held the hammerstone in their left hand; all others knapped right-handed.

### Ethics Statement

The experiments were approved by the University of Liverpool ethics committee (reference PSYC-1011-025 - Georg Meyer - Action planning and cerebral blood flow lateralisation). Written informed consent was acquired from all participants. All participants depicted this paper have given written informed consent, as outlined in the PLOS consent form, to publication of their images.

### Apparatus and Materials

**Figure 2:**
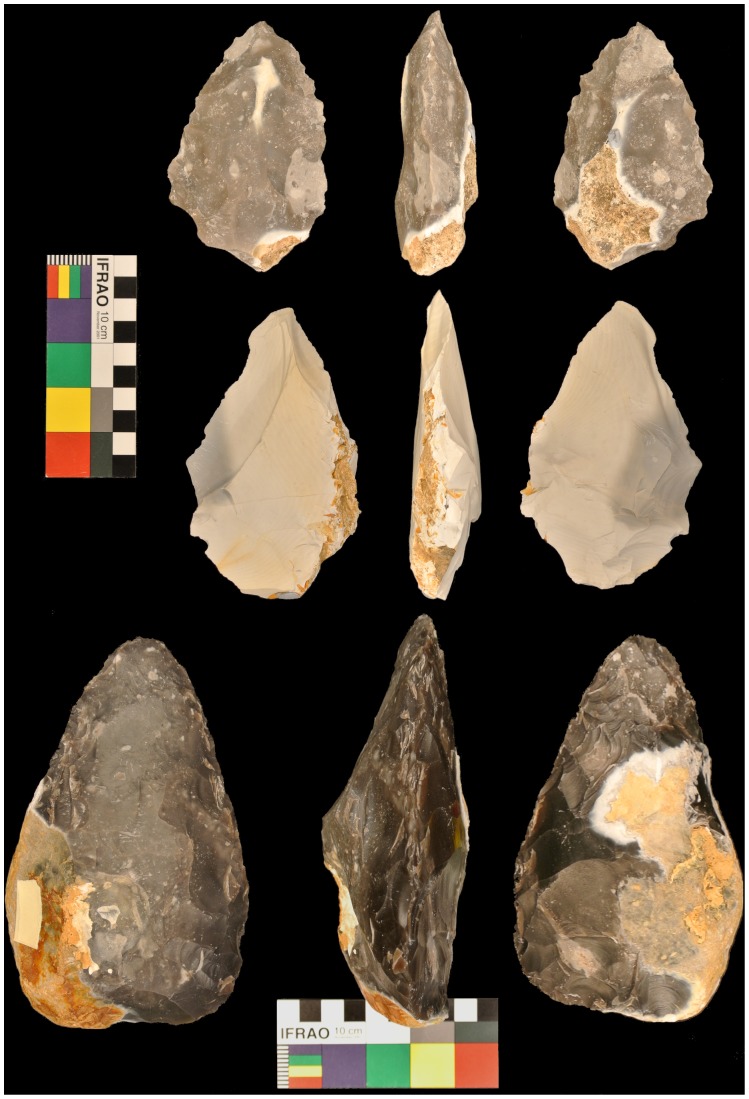
fTCD recording during the knapping task. A participant in our study carrying out the knapping task during fTCD recording. Two small, head-mounted probes measure cerebral blood flow velocities. The inset shows a diagram of the middle cerebral arteries that were insonated in our study.

Figure 2 shows a photograph of a participant carrying out the knapping task, and a schematic diagram of the fTCD setup. Blood flow velocity is simultaneously measured in both middle cerebral arteries (MCAs) using two headband mounted Doppler ultrasound probes [33]. Cerebral blood flow velocity (CBFV) in the right and left MCAs were continuously measured with a commercially available dual transcranial Doppler ultrasonography device (Multi-Dop T, DWL, Sipplingen, Germany). The MCAs were insonated at a depth of approximately 50 mm with two 2-MHz transducer probes attached to a headband and placed at the trans-temporal windows bilaterally [48]. The spectral envelope curves of the Doppler signals were recorded with a sample rate of 25 Hz.

### Experimental Conditions

We compare relative MCA blood flow velocity changes during two tasks designed to isolate the complex planning component of task execution: Acheulean handaxe production and silent cued word generation. In both experimental conditions, target intervals were alternated with control intervals. Following standard fTCD paradigms [28], [49], the target intervals were 25–35 seconds (s) (avg. 30 s) in duration while the control conditions were 15–25 s (avg. 20 s) long. Twenty target/control epochs were presented in each experimental block. Stimulus presentation was controlled by a personal computer running the ShowPics software (v. 3.1.0) which was interfaced to the fTCD system to mark the start of each epoch.

The cued word generation task, as discussed above, is a standard language lateralization assessment task used in clinical settings [42]. Subjects were asked to silently generate words starting with a letter heard at the onset of the target interval. Target letters were presented in random order and no letter was presented more than once. For the control interval subjects were asked to sit quietly and rest. A beep and a spoken letter marked the onset of the target interval while an isolated beep indicated the start of the control interval.

**Figure 3:**
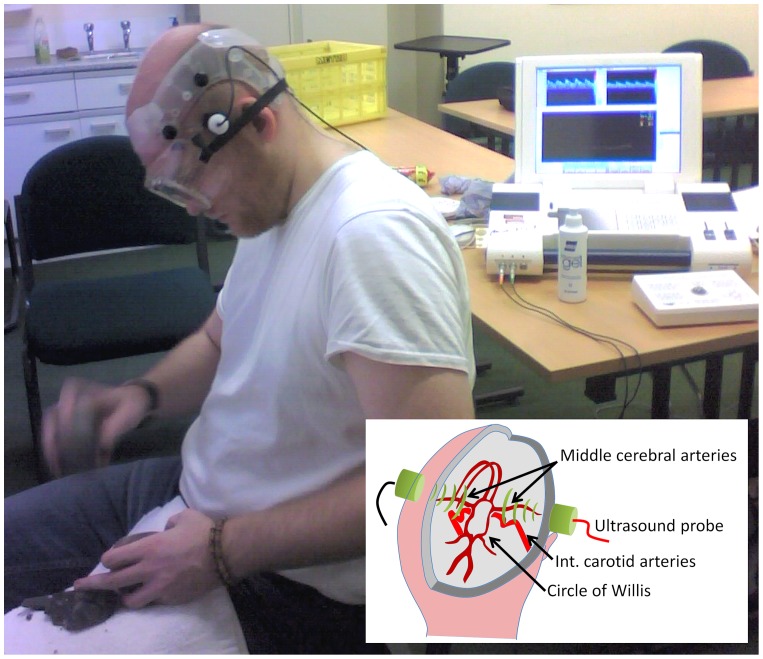
Stone tools produced in the experiment. Three handaxes produced by three participants in the experiment. Front, back, and side views are shown for each handaxe.

In the knapping task, subjects were asked to produce or continue producing a “generic” Acheulean handaxe in the target interval. This included manipulating the core, preparing the platform, and removing flakes by striking the hammerstone against the flint core. For the control interval, subjects were asked to keep hold of the same hammerstone and strike a large granite cobble with roughly the same intensity and frequency as in the knapping interval, but without trying to produce flakes or alter the shape of the stone. The Video S1 shows an example of one participant’s action and control intervals. Our paradigm was designed to isolate the action planning component during the target interval while keeping other activity, such as motor or visual processing, constant in both intervals. Thus, the action intervals differed from the control intervals in that subjects had to plan the flaking sequence necessary to produce a handaxe form. Subjects continued working on the same handaxe over a succession of target intervals. A beep generated by the controlling computer signaled a change in the intervals. Each participant had a total of 9 minutes of knapping time over the whole experiment. Figure 3 shows a sample of handaxes that were created by our participants.

Subjects were provided with a stone tool blank consisting of a large flake removed from the original quarried flint nodules of Brandon flint. Subjects selected a hammerstone from granite river cobbles provided for the experiment. During the experiment subjects had to knap with the same hammerstone and piece of flint, except in case of breakage or reduction to an unusable size. Subjects were provided with protective equipment, the test room was adequately ventilated during knapping, and the floor was protected by a tarpaulin for proper disposal of all knapping waste.

### Data Analysis

The recordings were integrated over the corresponding cardiac cycles, segmented into epochs and then averaged off-line using the AVERAGE V1.85 software [49]. Trials with physiologically implausible CBFV changes relative to baseline of +- 30% were excluded from the analysis. Subjects with less than 80% ‘good’ epochs in any one of the conditions were excluded from the data analysis (three of 13 subjects) to ensure data integrity. The average responses were filtered off-line using a second order zero-phase lag Butterworth low-pass filter with a cut-off frequency of 1 Hz.

All CBFV changes are computed relative to a baseline that was the average of the five seconds of the control period immediately preceding the target epoch onset. Group statistics were computed using purpose-designed MATLAB (The Mathworks, Natick, MA) scripts. The relative cerebral blood flow velocity Δ*V(t)* is the difference between left and right hemisphere blood flow velocity (Eqn.1):
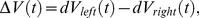
(1)where



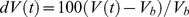
is the CBFV change relative to the mean baseline blood flow velocity (*V_b_*) recorded over the five seconds preceding the target condition onset.

The lateralization index (LI) represents the peak absolute lateralization value within the activation interval (Eqn. 2):
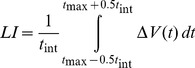
(2)


As integration interval, a time period of *t_int_* = 2 s was chosen. A positive value of the LI indicates left hemispheric processing dominance while negative values represent right hemisphere dominance.

## Results

**Figure 4:**
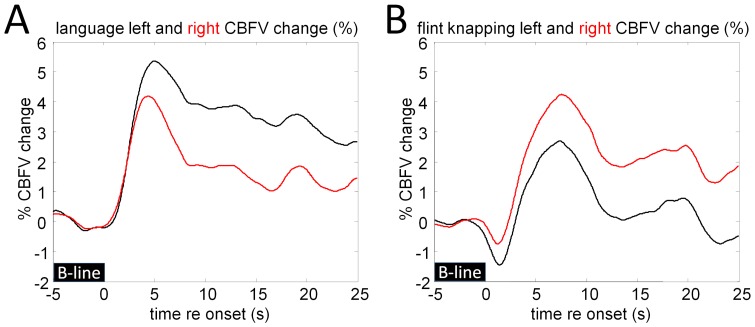
Bilateral CBFV changes during cued word generation and Acheulean tool-making. Bilateral CBFV changes were recorded simultaneously in the left (black trace) and right (red) MCAs for the language (A) and knapping (B) tasks. During the language task, blood flow in the left hemisphere increases more strongly than on the right, while the flint knapping task causes blood flow in the right artery to increase more than on the left. Blood flow changes are measured relative to a baseline, marked by a black bar (B-line), covering the mean CBFV over the final 5 seconds before task onset.

Figures 4A and 4B show the average CBFV changes during task execution in the left (black trace) and right (red trace) MCAs. During the language task (Figure 4A), blood flow in the left hemisphere increases more strongly than on the right. Peak blood flow changes are observed at around 5 s after task onset while the largest lateralization differences are seen after 7 s. Blood flow in the right hemisphere increases slightly faster immediately after the task onset. During the flint knapping task, we observe an initial brief dip in bilateral cerebral blood flow, followed by a relative increase in CBFV in both arteries (Figure 4B). CBFV in the right artery increases more than on the left with a pronounced peak in the average CBFV change around 7 seconds after task onset. After around 12 s, blood flow in the left MCA falls back to close to the baseline rate.

**Figure 5:**
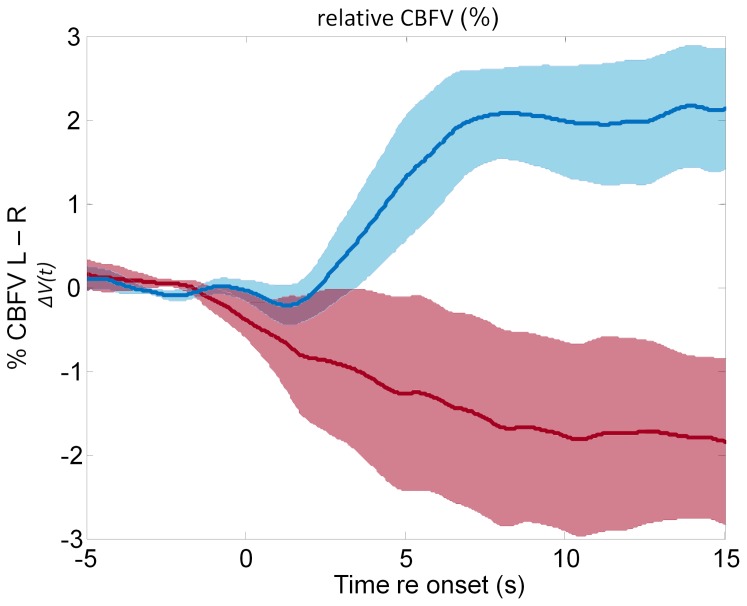
Brain blood flow volume lateralization changes for cued word generation and Acheulean tool-making. Raw mean CBFV (cerebral blood flow volume) difference (ΔV) over time during the language and knapping tasks. Positive values indicate a left dominant blood flow lateralization; negative values indicate greater blood flow on the right. The upper line shows the language task; the lower line shows the stone knapping task. The shaded areas show the SEM (standard error of the mean) at each data point.

Our fTCD recordings during the language task show a typical lateralization pattern (Figure 5). The direction, magnitude, and time course of CBFV changes as well as LI values reported here are consistent with previously reported data for the same task [28], [38], [46]. Between 10 s and 20 s after stimulus onset, subjects show an increase in average blood flow towards the left hemisphere (mean Δ*V* = 2.48%, sd = 2.38%, Δ*V* range: 1.21–6.29%) preceded by a small initial shift to the right (Figure 5, top trace). In the knapping task, the relative CBFV gradually shifts toward the right hemisphere (mean Δ*V* = −2.37%, sd = 4.08%, Δ*V* range: −9.33–+4.04%) over the initial 15 s of analysis (Figure 5, bottom trace). The mean CBFV lateralization during the stone knapping task is to the right hemisphere, consistent with previous findings of right dominant activations for experienced participants during stone knapping observation [50].

The main analysis focuses on rapid blood flow changes (ΔV) in order to assess our prediction of correlated blood flow changes for individuals during the initial phases of language and stone knapping. The conventional LI analysis [38] computes individual LI values as the average blood flow lateralization value in a 2-second window that is centered at the peak lateralization value. This means that for two different tasks the LI values can be computed for time windows in very different positions within the hemodynamic response. For the cued word generation and flint knapping tasks we show correlated LIs (r = 0.74, p = 0.013) in an analysis window between 2 and 18 seconds after task onset. However, for our analysis we imposed a further constraint by correlating only CBFV data from matching time windows in both responses, in order to test our prediction of a common modulation of hemodynamic responses if shared networks are used for both tasks.

**Figure 6:**
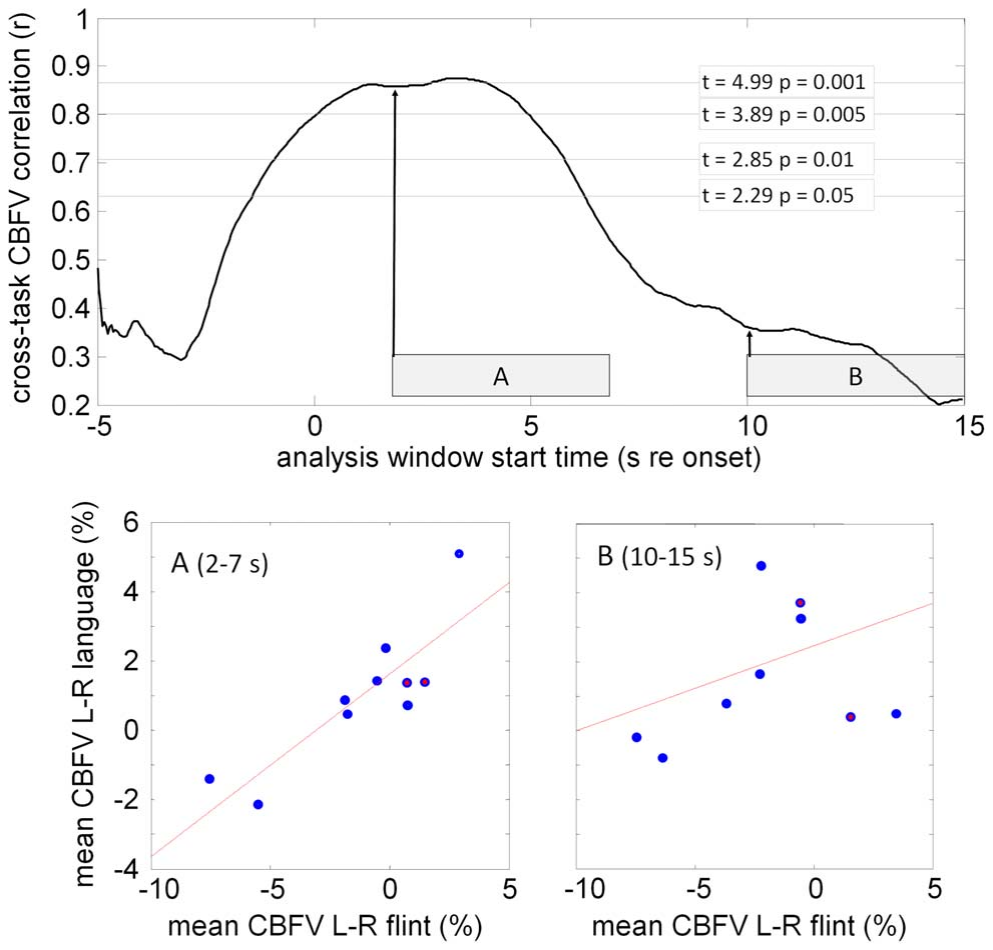
Correlation analysis of relative brain blood flow volume changes during language and knapping. The top panel shows the correlation between the mean CBFV differences (ΔV) for both tasks over a moving 5-second analysis windows. The x value represents the delay of the analysis window relative to the interval onset. Horizontal lines show uncorrected significance thresholds for the correlations. Bottom panels show individual subject data; the two left-handed subjects are represented by filled circles. The lines are linear fits. Window A covers 2–7 s after onset. Window B covers 10–15 s after onset.

Figure 6, top panel, shows the correlation coefficients for individual averaged CBFV differences (ΔV, Eqn. 1), computed over moving 5-second windows starting between 5 s before task onset (−5) and 15 s after the task onset. An analysis window of 5 s was chosen to match the time course of metabolic changes as measured with fMRI [51]. We show significantly correlated individual LIs for the two tasks for analysis windows starting between −2 and 7 seconds relative to signal onset (p<.05).

Figure 6, bottom panels, show the underlying correlation data for two 5-second windows starting 2 s and 10 s after task onset. The signals for the language and stone knapping tasks are significantly correlated in window A (2–7 s relative to task onset, r = 0.86, p = 0.0014). The correlation measures decline below the significance threshold for windows starting after approximately 7 s after task onset. At 10 s (window B) the correlation drops to r = 0.27 (p = 0.292).

## Discussion

Our participants showed correlated LIs during the initial 10 seconds after task onset for cued word generation and handaxe production. A number of previous studies have directly correlated LI values (Eqn. 2) for different tasks and shown that tasks which draw on shared neural processing sites, such as the three language tasks described by Bishop et al. [28], result in highly correlated LI measures. In contrast, cognitive tasks that draw on disparate brain areas, such as language and visuo-spatial tasks like driving [46], visual attention [44], or visual memory [45], lead to uncorrelated LIs. Since Acheulean stone knapping is a highly visuo-spatial task [30], [35], [52], our finding of a correlation between knapping and language requires explanation.

Making an Acheulean handaxe requires both working memory and planning memory [53]. This careful planning is dominant in the initial phase of each experimental block in our study. This action planning draws on brain areas that are shared with language tasks, such as the left-lateralized ventral premotor areas and Broca’s area [17], [18], [50]. Our subject pool shows highly correlated individual brain blood flow lateralization in the early phases of task execution for both tasks. Our findings add empirical data to the hypothesis that action planning for tool-making and language draw on shared functional brain structures [54], [55]. The correlated time-signatures for Acheulean knapping and language, which remain significantly correlated within subjects despite variability between subjects, indicates that the same brain networks are being activated for both tasks. They suggest that tool-making and language share a basis in more general human capacities for complex, goal-directed action.

The proposal that language and tool-making co-evolved is not new (e.g. [1], [9]–[11], [56]–[58]). Our findings are consistent with language evolution theories that posit a co-evolution of language and manual praxis [8], [11], [59]–[64]. Concurrent emergence of gestural and vocal communication would place a greater emphasis on the linkage of hand motor activity with linguistic networks. Among the many explanations for language origins [65], exaptation is one possibility. Co-evolution is another: the network for complex action planning might have emerged in human evolution as part of our brain size increase and reorganization [66], leading to both language and tool-making [1], [23], [57], [58], [61], [67]–[69]. We suggest the start of the Acheulean techno-complex at 1.75 million years ago [70] as a likely candidate for this because Acheulean knapping required more complex action planning than Oldowan technologies [14], [15], [31], [32], [50]. While the motor and visuo-spatial skills are the same for Oldowan and Acheulean knapping, these techno-complexes differ in the sequencing and hierarchical organization of the knapping gestures required [9], [10], [53], [71]–[74].

Whether the Acheulean techno-complex or language emerged first, or whether they emerged in parallel, cannot be resolved yet. However, following Tim Wakeford (pers. comm.), we propose that a co-emergence could explain the rapid and wide spread of the Acheulean, possibly due to improved teaching and learning of the knowledge and know-how for complex stone tool production facilitated by aspects of language [5], [30], [53], [62], [72], [75]–[78]. The Acheulean is remarkable for its combination of cultural conservatism *and* variability [79]–[84]. The social transmission of complex core reduction strategies used by hominins throughout the Acheulean world indicates that imitation and shared intentionality were already in place [85]. These could have been facilitated by proto-speech, proto-sign, or multimodal communication [86]–[88]. Future work should focus on specifying the extent of the potential left-hemisphere action-planning network, and on developing ways to neuroimage stone tool-making in real-time using techniques with good temporal and spatial resolution. Only after we can further dissect the temporal and spatial scales of action in stone knapping will we truly be able to make direct comparisons with language.

## Supporting Information

Video S1 **Video recording showing one participant knapping during the fTCD recording.** The equipment is visible in the background.
(WMV)
